# Consea is back! Or how to resist in challenging times

**DOI:** 10.1590/0102-311XEN086523

**Published:** 2023-06-26

**Authors:** Elisabetta Gioconda Iole Giovanna Recine

**Affiliations:** 1 Faculdade de Ciências da Saúde, Universidade de Brasília, Brasília, Brasil.

Important events could be listed in the “short period” of Brazil’s recent history, from January 1st, 2019, to February 28, 2023, in only one agenda, that of food and nutrition security.

When the *Provisional Measure n. 870/2019* ^1^ - which reorganized the structure of the previous Federal Government - was published, it contained what in practice was configured as the extinction of the Brazilian Food and Nutrition Security National Council (Consea). More than that, it caused the interruption of a complex but necessary process of implementing an intersectoral system of public policies responsible for articulating seemingly distant areas such as health, education, social development, economy, justice, environment, and culture, among others.

Since then, we have faced numerous challenges involving the weakening of public policies and institutions, the disruption of the social safety network, the impoverishment of the population, the precarization of work, and the global experience of the COVID-19 pandemic. As already discussed in the CSP’s editorial in 2019 ^2^, this provisional measure generated an important reaction for the return of Consea at the national and international levels. However, the situation had not been reversed.

In July, 2019, a set of organizations, movements, collectives and state councils of food and nutrition security involved with this agenda convened a National Popular Conference for Food and Nutrition Security and Sovereignty (CPSSAN). The convening document of this Conference reaffirmed the commitment to democracy, the fight against hunger and poverty, as well as to fight for all the policy instruments already conquered. They also declared that there would be no passivity in the face of what was foretold.

“*We, defenders of the human right to adequate food, stand firm, strong, and resilient and we will not surrender. We are in the cities, in the rural areas, in the forests, and in the waters, occupying spaces* (...) *and we are mobilizing society in favor of the agenda of sovereignty and food and nutritional security, in defense of democracy and for the establishment of rights*” ^3^ (free translation).

At that moment, we could not have foreseen the dimension that these words would assume in the following years. Originally, the CPSSAN stood as a process of mobilization that would culminate in a national meeting. With the pandemic, this meeting was not possible. Still, the commitment to mobilize and maintain the food and nutrition security agenda was established, possibly in an even broader way, with the different actions with the Brazilian National Congress for the protection of strategic programs, dissemination of emergency measures to protect the human right to adequate food during the pandemic.

The dialogue with society was also deepened. When the awful numbers for food insecurity were released by the *1st National Survey on Food Insecurity during the COVID-19 Pandemic in Brazil* (VIGISAN) ^4^, conducted in 2020, which identified 19.1 million people in a situation of hunger, the CPSSAN held a people’s court that condemned the Federal Government represented by the then chief of the executive branch for the hunger scenario in Brazil and handed down its sentence with a set of emergency actions ^5^. Although this sentence was attached to two actions that were in the Brazilian Supreme Court, we reached, at the beginning of 2022, 33 million people in hunger ^6^. In this same direction, a set of organizations, movements, formal and informal collectives, both well-established and recently formed, explicitly adopted the end of hunger as their fighting banner. Anti-racist, gender equality, indigenous, urban, peasant movements broadened their agendas, articulating political processes to confront poverty and hunger, which had been exacerbated by the pandemic. These initiatives originated actions of solidarity among equals, inspiring experiences that multiplied throughout the country in an evident sign echoing the “*we stand our ground, strong and resilient and we will not surrender*” call and led the popular field to strongly mobilize for the reversal of the political scenario.

The sum of losses accumulated in public policies for food and nutrition security in recent years cannot be attributed only to the lack of an institutional space for social participation, expressed by Consea. However, this absence certainly confirmed the impermeability of the Federal Government to recognize an evident situation and the importance of dialoguing with civil society to approach reality in a more qualified way and, from there, identify solutions ^7^.

During the 2022 electoral process, there was an intense mobilization of different popular sectors to influence their proposals in the delineation of programmatic priorities. As expected, the 33 million of people with hunger, extensive queues for bones, and food inflation populated newscasts and programs. Reality screamed even in the traditional media, but organized civil society demanded explicit commitments to overcome the situation. And, since they remained active during those years, they coherently acted throughout the transition period. The end of 2022 arrived loaded with expectations of all shapes and sizes, and everything in between, to return to the path of removing Brazil from the “Map of Hunger” and toward the return of Consea. And so it was, the *Provisional Measure n. 1,154/2023* ^8^, reinstalls Consea in the Presidency of the Republic and also recreates the Brazilian Ministry of Agrarian Development and Family Agriculture, revalues the human rights agenda and creates the Ministries of Racial Equality and Indigenous Peoples. There is no challenge that shall superimpose the evident sign that an era of overcoming and new challenges was starting.

On February 28 happened the ceremony for the reinstallation of the Consea, which returned with the same composition from before its extinction. Finally, we were able to declare the commitment to end hunger in Brazil and the need to articulate emergency actions with increased access to healthy food, in addition to promote measures toward cash transfer, job creation, combating racism and gender inequalities, and valuing the minimum wage. They also highlighted the urgency for a policy for supplying regulatory stocks, so that foods from peasant and family agriculture are accessible, considering that they are diversified and agroecological, respect food habits and cultures, and value biodiversity ^9^.

This ceremony is followed by the first plenary session of the Consea. The first two topics addressed the measures that were being taken to confront the humanitarian tragedy experienced by the Yanomami people and the presentation by the government of the initial outline of a broad strategy to overcome hunger. The experience of having overcome hunger in little more than a decade and having lost these results in a few years was the defining lines of the two strategic axes of the Consea: the defense of emergency measures to overcome hunger articulated with measures addressed to the roots of inequalities in Brazil and the overcoming of hunger with real food. Both guidelines have as transversal axes the collapse of climate, the anti-racist gender equalities struggle, and the defense of land and territory to whom it is entitled. This first plenary closes with the convening of the 6th National Conference on Food and Nutrition Security, which would have taken place in November 2019.

The conference will be responsible for defining the proposals to be sent to the Federal Government for the elaboration of the 3rd National Plan for Food and Nutrition Security. The urgency of a strategy that articulates different actions and sectors to confront hunger includes the need for a medium and long-term plan that enables transformations of the food system anchored in the concept of a global syndemic of malnutrition, obesity, and climate change ^10^. If these three great contemporary challenges share the hegemonic food system as a determinant, addressing these challenges requires a systemic approach to transformation. If determinants are shared, it is no longer enough to have a well-executed, but isolated, policy or program; it is necessary that the processes and results of one resonate and be enhanced with processes and results of other actions.

The trajectory of priorities expressed, for example, in the results of the five national conferences over the years is remarkable. Both the slogans that summoned them and their results always pointed to strategic advances and guided both their institutional and thematic future and contributed to the visibility of different social subjects ^11^. Consea houses an important diversity of social subjects that articulate their demands, knowledge, and practices in the same space, allowing for a democratic dialogue between differences and the achievement of agreements. The current moment, both national and global, requires the deepening of social representation and articulation. Policies and programs need to be addressed based on the issues and not on sectors (yes, I’m talking about intersectoriality!) so that we reach another level of thinking and making public policy, guarantee rights, and thus allowing us to deal with historical challenges with the knowledge of the present.
